# T70. RECOVERY IN FIRST –EPISODE PSYCHOSIS: COGNITIVE, CLINICAL AND RESILIENCE (PERSONAL RESOURCES) TRAJECTORIES ACROSS 6 YEARS

**DOI:** 10.1093/schbul/sby016.346

**Published:** 2018-04-01

**Authors:** Anne-Kari Torgalsboen, Christine Mohn

**Affiliations:** 1 University of Oslo; 2 Vestre Viken Hospital Trust

## Abstract

**Background:**

There is increasing recognition that people with schizophrenia can experience recovery. Based on a recent review of long-term outcome studies of first-episode psychosis (FEP), the authors argued that remission and recovery rates may be more favourable than previously thought.1 In order to improve timing of interventions and personalize treatment, it must be determined when improvement take place and how cognitive, clinical and resilience trajectories develop over time.

The Oslo Schizophrenia Recovery study is one of very few long-term prospective studies of FEP investigating the rate of recovery and the longitudinal course of cognitive, clinical and personal resources in the same study. Limitations of previous studies include high attrition and samples consisting of relapsing patients most often seen in inpatient settings. In the present study, fully recovered subjects no longer in treatment have not been lost to follow- up.

The present study has a multi-follow-up design, is ongoing, and includes data from eight assessment points across six years. Thus, it is possible to assess sustained remission and full recovery as well as to show trajectories of neurocognition, symptoms and personal resources in the long term.

**Methods:**

28 (17 men, 11 women, mean age 21.0, SD 2.6 years) individuals with first-episode schizophrenia and receiving a combination of medication, case management and cognitive therapy are assessed with the Positive and Negative Syndrome Scale (PANSS), the MATRICS Consensus Cognitive Battery (MCCB) and measures of self-efficacy, hope and resilience at each assessment point. Repeated measures ANOVAs were conducted.

**Results:**

At 6-year follow-up 45.5 % fulfilled criteria for full recovery, i.e. sustained improvement in both symptoms and social/vocational functioning for two years or longer. The retention rate is high (79%). As expected, there were statistically significant reductions in both positive and negative symptoms from baseline, with stabilization from 2 years to 6 years follow-up: F 21.77, p .000, η2 .53 and F 46.60, p .000, η2 .71. The same significant reduction across 6 years was shown for general psychopathology (F 31.36, p .000, η2 .62.) There were statistically significant increases in overall neurocognitive test results from baseline, with stabilization from 2 years to 6 years follow-up: F 11.25, p .000, η2 .35.

A significant increase in reported hope was found from baseline with stabilization from 6 months to 6 years follow-up: F 5.53, p .000, η2 .21. The trajectory of general self-efficacy followed almost the same pattern as hope: There were statistically significant increases in reported self-efficacy from baseline, with stabilization from 1 year to 6 years follow-up: F 3.37, p .002, η2 .14. Finally, resilience also increased significantly from baseline, with stabilization from 6 months to 6 years follow-up: F 5.12, p .000, η2 .21.

**Discussion:**

The recovery rate in this sample is higher (45.5%) compared to other studies. Modern treatment, as well as keeping the fully recovered in the study, may all contribute to this higher rate of recovery. The results indicate that the greatest improvement happens during the two first years of treatment and thus seems to represent a “window of opportunity” for recovery. Increases in reported self-efficacy, hope and resilience were observed 1., 5 years before improvements in neurocognition and clinical symptoms, underlining the importance of focusing on personal resources and patients’ hope of recovery early in treatment with the ultimate aim of increasing the rate of recovery in FEP.

**References:**

1. Lally, J., et al. Remission and recovery from first-episode psychosis in adults: systematic review and meta–analysis of long –term outcome. British Journal of Psychiatry, 2017.

